# Evidence for a Tumor-Suppressive Role of SHP-1 in EMT Regulation in Bladder Cancer Cells

**DOI:** 10.3390/cancers18091401

**Published:** 2026-04-28

**Authors:** Kailey Hooper, Shannon McNall, Daniel Pohl, Travis Sullivan, Eric Burks, Kimberly Rieger-Christ

**Affiliations:** 1 Department of Translational Research, Lahey Hospital & Medical Center, Burlington, MA 01805, USA; kailey.e.hooper@lahey.org (K.H.);; 2 Department of Urology, Lahey Hospital & Medical Center, Burlington, MA 01805, USA; 3 Department of Pathology, Boston University Chobanian & Avedisian School of Medicine, Boston, MA 02118, USA

**Keywords:** bladder cancer, SHP-1, PTPN6, tumor suppressor, pAkt, epithelial–mesenchymal transition

## Abstract

**Background/Objectives:** Src homology 2 domain-containing protein tyrosine phosphatase 1 (SHP-1), also known as protein tyrosine phosphatase non-receptor type 6, functions as a tumor suppressor in breast, hepatocellular, and prostate cancers and an oncogene in glioblastoma and cervical cancer. A previous analysis of The Cancer Genome Atlas (TCGA) dataset revealed that lower SHP-1 transcript levels in bladder tumors were associated with poorer overall survival. **Methods:** This study aimed to evaluate the role of SHP-1 in bladder cancer and to assess the functional impact of its forced expression and knockdown in bladder carcinoma cell lines. SHP-1 expression was assessed in 19 bladder cancer cell lines and 26 bladder tissues. Lentiviral transduction was used to knock down or overexpress SHP-1 in four cell lines, followed by Western blot analysis of SHP-1 and pAkt/Akt protein expression. **Results:** SHP-1 protein levels were significantly lower in highly invasive cell lines (*p* < 0.001) and muscle-invasive tumors (*p* < 0.05). Functional studies demonstrated that SHP-1 modulation influenced the epithelial–mesenchymal transition (EMT) phenotype. SHP-1 expression was positively correlated with E-cadherin expression (*p* < 0.001) and negatively correlated with N-cadherin (*p* < 0.01) and Vimentin (*p* < 0.05) expression. Alteration of SHP-1 expression in bladder cancer cell lines affected proliferation, invasion, and migration (*p* < 0.05). RNA-seq analysis of the transduced cell lines revealed enrichment of gene sets related to EMT and signaling pathways involving MYC, PI3K, Akt, and mTOR. Furthermore, SHP-1 alteration impacted pAkt/Akt ratios (*p* < 0.05). **Conclusions:** Collectively, lower SHP-1 protein expression correlated with more aggressive phenotypes in bladder cancer cell lines and bladder tumors. In our limited dataset, reduced SHP-1 expression correlated with muscle-invasive disease, suggesting a potential link to more advanced tumor biology, consistent with TCGA associating reduced SHP-1 transcript expression to poorer survival rates. Our data provide preliminary functional evidence that SHP-1 may modulate Akt signaling in bladder cancer. Together, these results support further investigation of SHP-1 as a possible tumor suppressor, candidate prognostic biomarker, and potential therapeutic target in bladder cancer.

## Simple Summary

Bladder cancer is a common and costly malignancy with high recurrence rates and variable survival outcomes, highlighting the need for improved molecular targets. Src homology region 2 domain-containing phosphatase-1 (SHP-1) has been identified as a potential tumor suppressor in several cancers, and its reduced expression in bladder cancer has been associated with poorer survival outcomes. However, its role in bladder cancer remains poorly understood. Therefore, this study focused on SHP-1, aiming to evaluate its potential contribution to bladder cancer progression and to assess whether it may represent a viable target for future therapeutic strategies.

## 1. Introduction

Bladder cancer (BCa) represents a significant health burden, with over 84,530 new diagnoses and more than 17,870 deaths estimated in the United States for 2026 [1]. BCa is the sixth most common malignancy in the United States, with urothelial carcinomas comprising over 95% of cases [2]. Although BCa incidence in the United States has gradually decreased over the past 10 years at a rate of 1.1% a year [3], it continues to be one of the costliest malignancies in the healthcare system [4]. This cost is due in part to the highly recurrent nature of BCa, with a recurrence rate of 42–67% [5] for muscle-invasive and 70–80% [6] for non-muscle-invasive BCa. The overall 5-year survival rate for in situ BCa is 98%, that for localized BCa is 71%, that for regional BCa is 39%, and that for metastatic BCa is 8%, with an overall relative survival rate of 79% [3]. While targeted therapeutic approaches are increasingly being incorporated into BCa management, a critical need remains for improved molecular characterization to define the molecular dysregulation contributing to disease progression and to identify actionable therapeutic targets.

BCa development and progression are strongly influenced by the dysregulation of several tumor suppressor pathways. Of these, the TP53 pathway is the most well characterized, where mutations frequently occur in muscle-invasive BCa. Its dysregulation contributes to impaired DNA damage responses and apoptotic signaling [7,8]. It often coincides with mutations in RB1, driving proliferation, and they are collectively associated with poor patient outcomes [7,9]. Moreover, several studies have highlighted the importance of tumor suppressors involved in chromatin remodeling and epigenetic regulation. This includes genes such as KDM6A and ARID1A, which influence transcriptional regulation and differentiation in BCa development [10,11]. Furthermore, in BCa, there is often dysregulation of the PI3K/Akt signaling pathway through loss of PTEN, or alterations in pathway components. This leads to enhanced tumor cell survival, proliferation, and metabolic reprogramming [7,12]. The disruption of these interconnected suppressive networks collectively contributes to urothelial transformation, and this plays a major role in determining the tumor phenotype, aggressiveness, and therapeutic response.

Src homology region 2 domain-containing phosphatase-1 (SHP-1), also known as *PTPN6*, is a tyrosine phosphatase signaling molecule that has been found to serve as a tumor suppressor in several malignancies such as prostate cancer [13], hepatocellular cancer [14], breast cancer [15], lung cancer [16], colorectal cancer [17], and esophageal cancer [18]. In general, protein tyrosine phosphatases function as signaling molecules in the regulation of various cellular processes such as protein phosphorylation, cytokine production, cell growth, cell differentiation, and the mitotic cycle [19]. Outside of BCa, effects of SHP-1 on downstream targets such as MAPK [20], SRC [15], STAT3 [21], and Akt [22] have been reported, and it has also been implicated as a negative regulator of epithelial–mesenchymal transition (EMT) [14,22].

Analyses of SHP-1 expression in BCa based on The Cancer Genome Atlas (TCGA) dataset revealed that reduced transcript levels in tumor samples correlated with significantly poorer overall survival, potentially implicating a role for SHP-1 in BCa pathogenesis [23,24]. In addition, an in vitro study using four distinct BCa cell lines demonstrated that SHP-1 promoted apoptosis, thus suggesting a functional role of SHP-1 in restraining tumor growth [25]. Collectively, these findings support the premise that SHP-1 may function as a tumor suppressor in BCa. However, its role in this disease remains poorly defined, as existing data are largely limited to expression-based associations, and a relationship between SHP-1 protein expression and clinically relevant features such as muscle-invasive progression has not been reported. Moreover, detailed mechanistic studies delineating how SHP-1 modulates oncogenesis in BCa remain limited, underscoring the need for further investigation.

Therefore, this study aimed to investigate a potential functional and mechanistic role of SHP-1 in BCa, including a preliminary examination of its association with aggressive disease features. Here, we aimed to assess the role of SHP-1 in BCa by examining its expression in bladder tissues and BCa cell lines and evaluating its association with EMT and the metastatic phenotype. In addition, we assessed the functional effects of SHP-1 on tumor cell proliferation, migration, and invasion. Furthermore, we sought to investigate the mechanism by which SHP-1 affects BCa cellular activity by examining the impact of differential SHP-1 expression on downstream targets.

## 2. Materials and Methods

### 2.1. Cell Culture

Human BCa cell lines 5637, BC16.1, CUBIII, EJ, HT-1376, Hu456, J82, KK-47, LUCC2, MGH-U1, PSI, RT-112, RT-4, SW780, T24, TCC92-1, TCCSUP, UM-UC-3, and UM-UC-14 (ATCC and kind gifts from Dr. C. Reznikoff) were cultured under standard conditions (37 °C, 5% CO_2_) in Dulbecco’s Modified Eagle Medium (ATCC, Manassas, VA, USA) supplemented with 10% fetal bovine serum, penicillin/streptomycin, and L-glutamine. The cell lines were treated prophylactically for mycoplasma using BM-Cyclin (Sigma-Aldrich, St. Louis, MO, USA) once thawed and monitored routinely by DAPI staining but were not independently authenticated.

### 2.2. Lentiviral Transduction of Bladder Cancer Cells

#### 2.2.1. SHP-1 Knockdown

The high-SHP-1-expressing cell lines CUBIII and RT112 were utilized for SHP-1 knockdown studies. Lentiviral shRNA constructs targeting SHP-1 were obtained from Origene (Rockville, MD, USA), and the manufacturer’s protocol was used to establish stable cell lines for each construct. Each cell line was transduced with a SHP-1 shRNA construct, with unique sequences used for each cell line (CUBIII: TL310057VB-AGCCAGCCTGGAGACTTCGTGCTTTCTGT and RT112: TL310057VD-CACAAGGAGGATGTGTATGAGAACCTGCA), as well as a negative control (TR30021V scramble control—GCACTACCAGAGCTAACTCAGATAGTACT) in both lines. Transduced cell lines were maintained in medium with puromycin selection agent. All cell assays were performed in medium devoid of selection agent. The shRNA knockdown of SHP-1 was verified via Western blot analysis.

#### 2.2.2. SHP-1 Rescue

The low-SHP-1-expressing cell lines TCCSUP and UM-UC-3 were utilized for SHP-1 rescue studies. Lentiviral-based SHP-1 constructs (LPP-H1802-LV105-100S and the corresponding negative control) were obtained from Genecopeia (Rockville, MD, USA), and the manufacturer’s protocol was used to establish stable cell lines with recombinant SHP-1 protein expression. Each of the transduced lines was maintained in medium with puromycin selection agent. All cell assays were performed in medium devoid of selection agent. SHP-1 rescue was verified via Western blot analysis.

### 2.3. Cell Proliferation Assay

Cells were plated into a Corning^®^ Falcon^®^ 96-well white flat-bottom plate (353296, Corning Costar Corporation, Cambridge, MA, USA) at a density of 1 × 10^4^ cells/mL. The density of the cells was determined using a hemocytometer (American Optical Corporation, Buffalo, NY, USA). All conditions were tested. Each condition was tested in duplicate per experiment, and the experiments were independently repeated on multiple days. Forty-eight (48) h after culturing, a 1:2000 dilution of RealTime-Glo™ MT Cell Viability Assay (Promega, Madison, WI, USA) reagents and respective media were added to each well. Luminescence readings were produced by utilizing a GloMax^®^ Explorer Multimode Microplate Reader (Promega, Madison, WI, USA) following 1 h incubation with the Cell Viability reagents.

### 2.4. Cell Migration and Invasion Assays

Modified Boyden chamber assays were used to evaluate in vitro migration and invasion. Transwell membrane filter inserts (8 μm pores, Corning) were placed in 24-well plate reservoirs. For migration assays, cells were seeded at 1 × 10^4^ cells/mL. Migration assays were conducted by placing chambers in wells filled with serum-free medium with fibronectin (10 μg/mL). Cells were allowed to migrate toward the underside of the membrane for twenty-four hours at 37 °C.

For the invasion assay, Corning^®^ BioCoat™ Matrigel^®^ Invasion Chambers (354480, Corning Costar Corporation, Cambridge, MA, USA) were used. Cells were seeded at 1 × 10^4^ cells/mL in serum-free media onto the upper surface of the membrane and allowed to invade toward the underside of the membrane for twenty-four hours at 37 °C.

After incubation, cells that migrated or invaded through the membrane were fixed in 10% *w*/*v* neutral-buffered formalin (Simport, Beloeil, QC, Canada) and stained with DAPI (Invitrogen, Carlsbad, CA, USA) (1:500 dilution in PBS, 1% Triton X-100). Cell counts for all migration and invasion assays were obtained using a fluorescence microscope (Evos; Advanced Microscopy Group, Bothwell, WA, USA). Three unique image frames per well and three wells per individual experiment were counted.

For all cell assays, each condition was evaluated in a minimum of three independent experiments using cells from separate passages, which were treated as biological replicates. Technical replicates within each experiment (i.e., duplicates or triplicates) were averaged to generate a single value for subsequent analysis.

### 2.5. Western Blot Analysis

Protein lysates were prepared from cells at 70–80% confluency. Cells were lysed directly in culture dishes using 200 μL/well of boiled 1× SDS-Laemmli (250 mM Tris-HCl, 4% SDS, 10% glycerol, 0.003% bromophenol blue), with lysates collected by manual scraping using a cell scraper and further sheared with a 24-gauge needle. Protein concentrations were determined via a BCA assay (Pierce, Rockford, IL, USA). Samples were normalized to a total protein concentration of 40 µg/mL using ProteinSimple sample buffer containing DTT and heated at 95 °C for 5 min; then, the samples were dispensed into individual wells of a 12–230 kDa separation module on the Simple Western Jess system (ProteinSimple, Santa Clara, CA, USA). Before use, SHP-1 primary antibody (AF1878, R&D Systems, Minneapolis, MN, USA) was diluted 1:20 in Antibody Diluent 2 (ProteinSimple); E-cadherin primary antibody (MAB1838, R&D Systems, Minneapolis, MN, USA) was diluted 1:200 in Antibody Diluent 2; N-cadherin primary antibody (ab18203, Abcam, Waltham, MA, USA) was diluted 1:200 in Antibody Diluent 2; Vimentin primary antibody (AF2105, R&D Systems) was diluted 1:25 in Antibody Diluent 2; total Akt primary antibody (9272, CST, Danvers, MA, USA) was diluted 1:100 in Antibody Diluent 2; and phospho-Akt (S473) pan-specific primary antibody (AF887, R&D Systems) was diluted 1:50 in Antibody Diluent 2. Anti-goat (DM-006, ProteinSimple), anti-mouse (DM-002, ProteinSimple), or anti-rabbit (DM-001, ProteinSimple) secondary antibodies were used as appropriate, according to the manufacturer’s protocol. Normalization of SHP-1 values against total protein expression was conducted in each corresponding capillary, using RePlex and Total Protein Detection reagents (ProteinSimple). Each condition was evaluated as an independent experiment by using cell lysates from separate passages, which were treated as biological replicates. Each sample was tested in duplicate, and these values were averaged to obtain a single value for subsequent analysis.

### 2.6. Patient Material Characterization

Under an Internal Review Board-approved study, tissues were prospectively collected at the time of surgery from 40 male patients undergoing cystectomy and flash-frozen in liquid nitrogen. This study is a retrospective analysis of these archived specimens. Patients included in this study had urothelial carcinoma on final pathology, with or without squamous or glandular differentiation. Patients were excluded if they had received neoadjuvant chemotherapy, had evidence of metastatic disease, or had final pathology demonstrating a non-bladder primary malignancy or non-urothelial bladder cancer. Representative H&E sections of each frozen tissue specimen were reviewed by a pathologist specializing in urologic malignancies (EB) to verify tissue status: either urothelium with no residual disease (NRD) or tumor tissue with a high percentage of tumor nuclei. This frozen tissue was used to assess SHP-1 protein expression, whereas tumor grade and stage were assigned based on the pathology report of the corresponding resected FFPE specimen.

### 2.7. Immunohistochemistry of Frozen Sections

Immunohistochemistry of frozen sections (IHC-F) was performed on bladder tissue using a HRP/DAB detection kit (ab64264, Abcam), following the manufacturers’ recommended protocol. Briefly, tissue was embedded in OCT, cut into 5 µm sections, mounted on charged glass slides, and fixed in methanol. After blocking and peroxidase treatment, the tissue sections were incubated overnight in antibody diluent at 2–8 °C, with SHP-1 primary antibody (AF1878, R&D Systems) used at 1:100. After detection with the kit’s secondary antibody/HRP and DAB, the tissues were counterstained with hematoxylin.

### 2.8. QuPath Analysis of Frozen Sections

Slides were imaged with a 20× objective using a Leica DMi8 equipped with a DMC2900 camera through the Leica LAS X microscope software v3.7.6.25997 (Leica Microsystems CMS GmbH, Wetzlar, Germany), yielding a resolution of approximately 0.3 µm/pixel. Quantitative analysis of the DAB staining was performed using QuPath software v0.3.2 [26]. Cell detection was performed using the hematoxylin OD settings, a background radius of 10 µm, and a cell expansion of 10 µm. The DAB vector was refined using the “Estimate stain vectors” command to accurately distinguish hematoxylin from DAB across all images. Positivity was determined based on the Cell: DAB OD mean with a single threshold setting of 0.1. This binary approach was chosen over multi-threshold settings to minimize the impact of the non-linear properties of DAB staining. The output for analysis was the percentage of positive cells within each entire region of interest.

### 2.9. Statistical Analysis

Statistical analyses were performed on biological replicate data using SPSS v29.0.1.0 (IBM). Continuous variables were assessed for normality using the Shapiro–Wilk test. For comparisons between two groups, Welch’s t-test was used, as there was no evidence of deviation from normality. For the comparison among three groups (IHC analysis), the assumption of normality was not met, and the Kruskal–Wallis test was used. When the results of the Kruskal–Wallis test were significant, post hoc comparisons were conducted with Bonferroni correction to adjust for multiple comparisons. Categorical variables were analyzed using Fisher’s Exact test. Charts were prepared in R (v4.5.2) using the ggplot2 package.

### 2.10. RNA Library Preparation and Sequencing

Total RNA was isolated from the four cell lines with SHP-1 transduction phenotypes (knockdown and rescue), as well as the corresponding negative controls. NGS RNA gene expression analysis was performed by Genewiz (South Plainfield, NJ, USA) on 16 samples: each of the eight transduced cell lines were analyzed twice from separate passages, which were treated as biological replicates. The libraries for RNA sequencing were constructed with a NEBNext Ultra II kit for Illumina, following the supplier’s guidelines (NEB, Ipswich, MA, USA). In brief, enriched RNA was first subjected to thermal fragmentation at 94 °C for 15 min. This was followed by first- and second-strand cDNA synthesis. The resulting cDNA fragments underwent end repair and 3′ adenylation, and then universal adapters were ligated. Index sequences were then incorporated, and the libraries were amplified using a limited number of PCR cycles. Library quality was assessed with an Agilent Tapestation 4200 (Agilent Technologies, Palo Alto, CA, USA), and concentrations were measured using a Qubit 4 Fluorometer (ThermoFisher Scientific, Waltham, MA, USA) and quantitative PCR (KAPA Biosystems, Wilmington, MA, USA).

The prepared libraries were pooled for multiplexing and distributed across four flow cell lanes. Following this, the flow cell was run on an Illumina HiSeq 4000 platform according to the manufacturer’s protocol. Sequencing was carried out in a 2 × 150 bp paired-end format. The resulting raw data files (.bcl) generated by the instrument were subsequently converted into FASTQ files and demultiplexed with Illumina bcl2fastq software (v2.20), allowing for a single mismatch during index assignment.

### 2.11. NGS Data Analysis

Following demultiplexing, sequencing output was evaluated for overall quality metrics and yield. Adapter contamination and low-quality bases were removed using Trimmomatic (v.0.36). The cleaned reads were aligned to the human GRCh38 reference genome obtained from ENSEMBL using the STAR aligner (v.2.5.2b). The gene-level counts were generated with feature Counts in the Subread package (v.1.5.2), considering only uniquely aligned reads mapping to the exon regions. Gene Set Enrichment Analysis (GSEA; v.4.3.3) [27] was performed to determine whether gene sets were significantly enriched for genes ranked by differential expression between the SHP-1 expression conditions (higher SHP-1: CUBIII shNC, RT-112 shNC, TCCSUP +SHP-1, and UM-UC-3 +SHP-1 versus lower SHP-1: CUBIII shSHP-1, RT-112 shSHP-1, TCCSUP +NC, and UM-UC-3 +NC). The genes were ranked by log2 fold change, and enrichment scores were calculated using the hallmark gene set collection (v2024.1) to determine overrepresentation. Normalized enrichment scores were utilized to account for overall differences in gene set size, and the resulting *p*-values were adjusted for multiple comparisons to control the false discovery rate (FDR). Given the exploratory, hypothesis-generating nature of this part of the investigation, an FDR < 0.1 was used.

## 3. Results

### 3.1. SHP-1 Expression Is Downregulated in Mesenchymal-like Cell Lines and Muscle-Invasive Bladder Cancer Tissue

**Figure 1:**
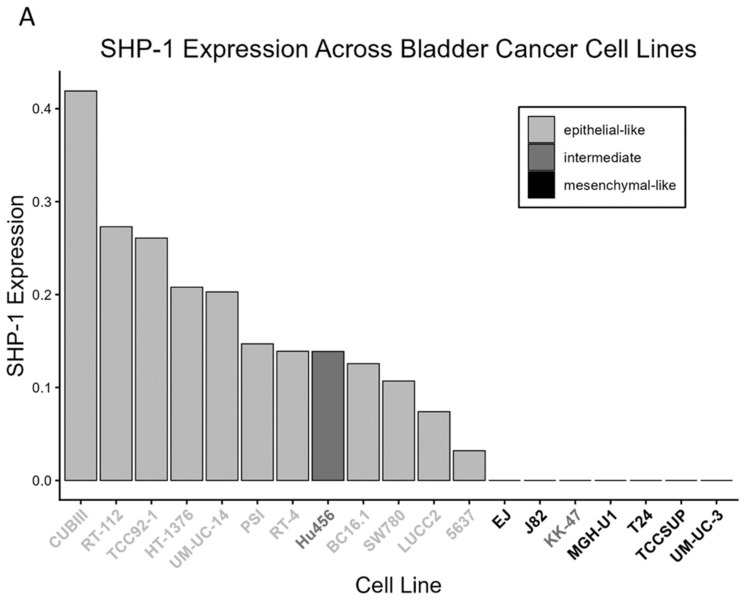

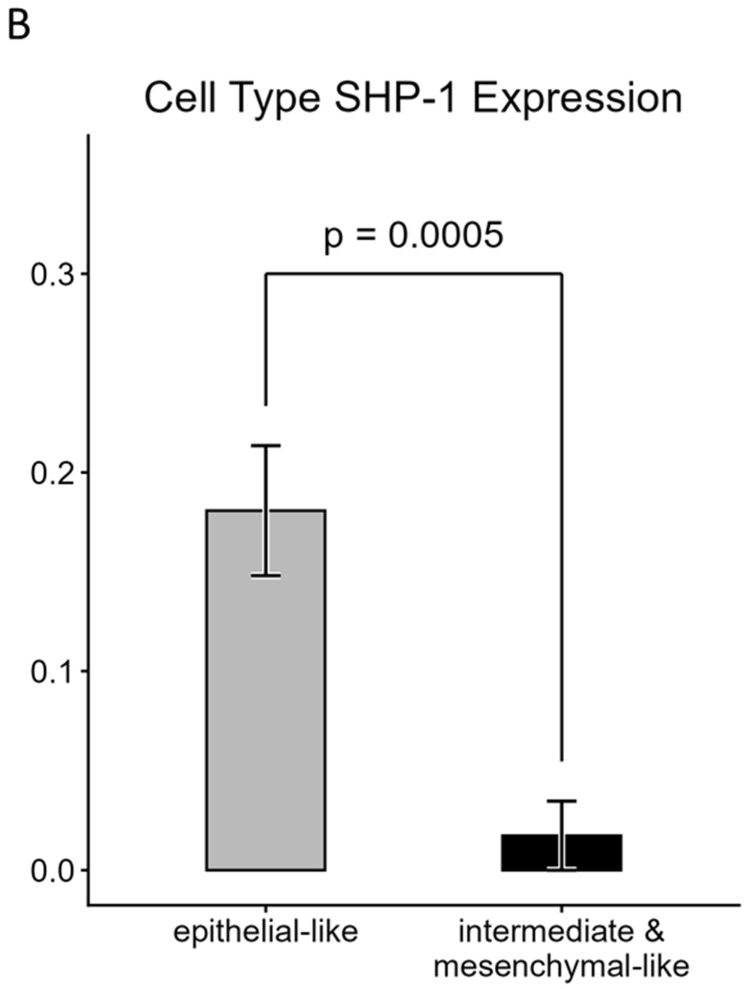
(**A**) Relative SHP-1 protein expression in the 19 BCa cell lines examined in this study. (**B**) Expression levels were significantly higher in epithelial-like cells (n = 11) than in intermediate and mesenchymal-like lines (n = 8). Error bars represent the standard error of the mean (SEM).

To characterize the expression profile of SHP-1 in BCa, we first screened 19 established BCa cell lines via Western blot analysis (Figure 1A). Based on established phenotypic classifications [28,29,30,31,32,33], the cells were categorized as epithelial-like, intermediate, or mesenchymal-like. SHP-1 expression was significantly higher in the epithelial-like cell lines (e.g., CUBIII and RT-112) than in the intermediate and mesenchymal-like lines (e.g., KK47 and UM-UC-3) (*p* = 0.0005; Figure 1B). For each cell line, one biological replicate was tested in duplicate, and the average value is presented.

**Figure 2:**
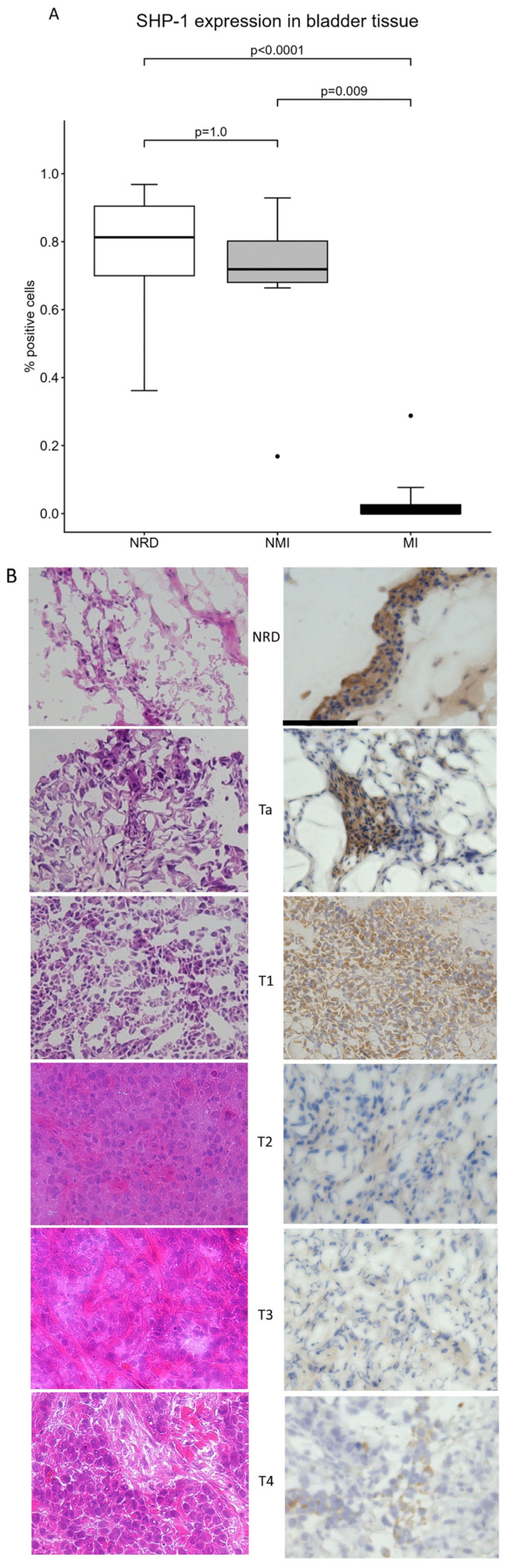
(**A**) SHP-1 protein expression in bladder tissue was significantly lower in muscle-invasive (MI, n = 8) tumor than in non-muscle-invasive (NMI, n = 8) and urothelial tissue with no residual disease (NRD, n = 10). (**B**) Examples of DAB and corresponding H&E staining in the bladder tissues examined in this study. The error bars represent the interquartile range.

To validate these findings in clinical specimens, we performed IHC on 26 of 40 frozen bladder tissue samples that met the inclusion and exclusion criteria. Fourteen of the tissues were excluded because of inadequate viable tumor cell content or, in the case of NRD, insufficient urothelium. Patient demographics are shown in Table 1. The median percentage of SHP-1-positive cells was highest in urothelial tissue with no residual disease (NRD, median = 0.81) and non-muscle-invasive BCa (NMI ≤ T1, median = 0.72), with no significant difference between these two groups. However, the muscle-invasive BCa samples exhibited a significant loss of SHP-1 expression (MI ≥ T2, median = 0; Figure 2A) compared to both the NRD and NMI groups, suggesting that SHP-1 downregulation is associated with advanced disease stage. These results should be interpreted with caution, as the NRD specimens were used as a surrogate for normal urothelium, and the NRD tissue may contain molecular traces related to the prior disease. Examples of the IHC are shown in Figure 2B.

### 3.2. SHP-1 Expression Correlates with an Epithelial Phenotype

**Figure 3:**
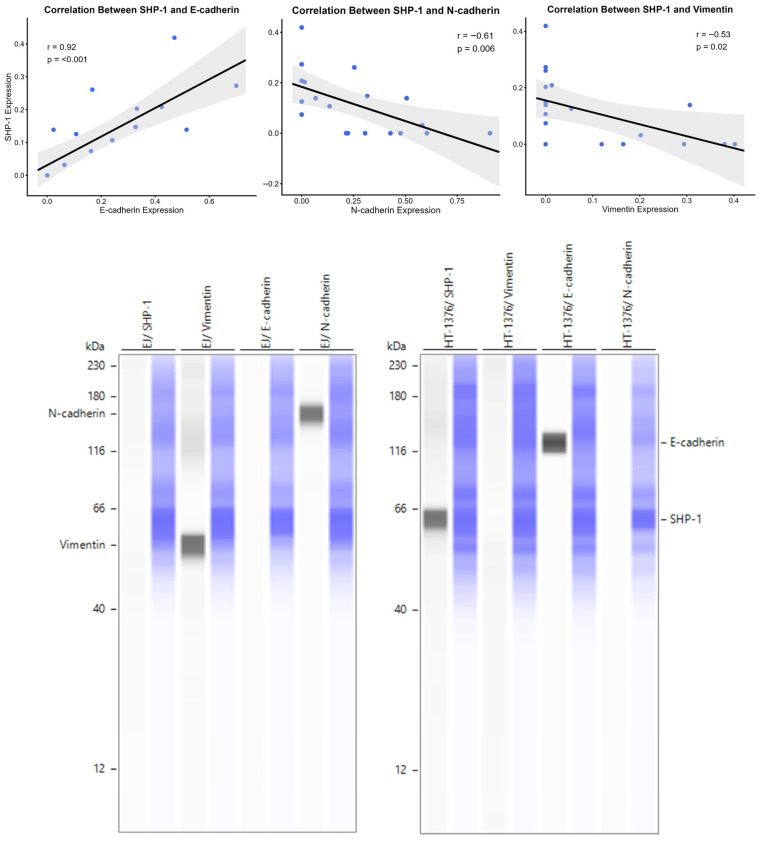
Correlation plots (n = 19) of SHP-1 protein expression with the EMT markers E-cadherin, N-cadherin, and Vimentin. Gray areas represent the 95%CI. Representative Western blot images: from the BCa cell lines EJ and HT-1376, the first lane of each pair corresponds to the protein indicated above each lane, and the second corresponds to the total protein detected for the same lane.

Given the differential expression across cell line phenotypes, we investigated the correlation between SHP-1 and classical EMT markers. Spearman’s rank correlation analysis using protein expression from each of the 19 cell lines revealed that SHP-1 expression is strongly positively correlated with the epithelial marker E-cadherin (rho = 0.92, *p* < 0.001). Conversely, SHP-1 expression was significantly negatively correlated with the mesenchymal markers N-cadherin (rho = −0.61, *p* = 0.006) and Vimentin (rho = −0.53, *p* = 0.02; Figure 3). For each cell line, one biological replicate was tested for each protein in duplicate, and the average value is presented.

### 3.3. SHP-1 Regulates Bladder Cancer Cell Proliferation, Migration, and Invasion

**Figure 4:**
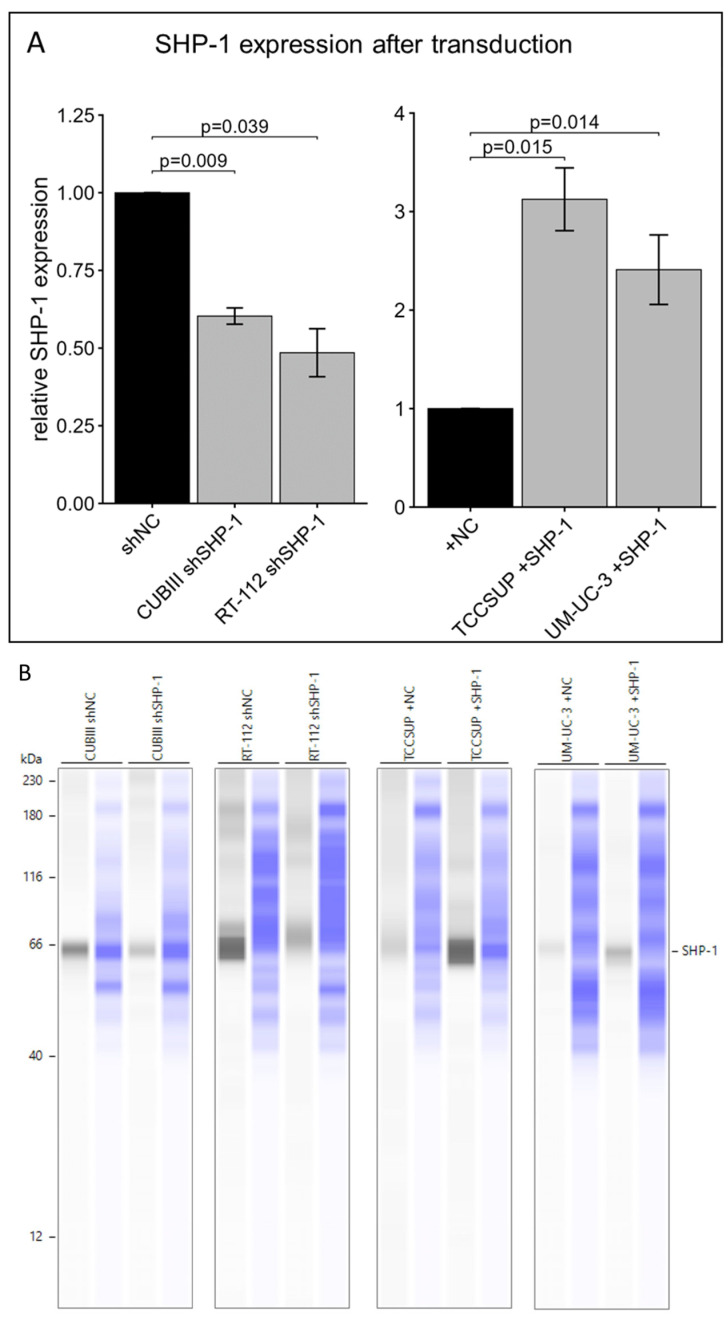

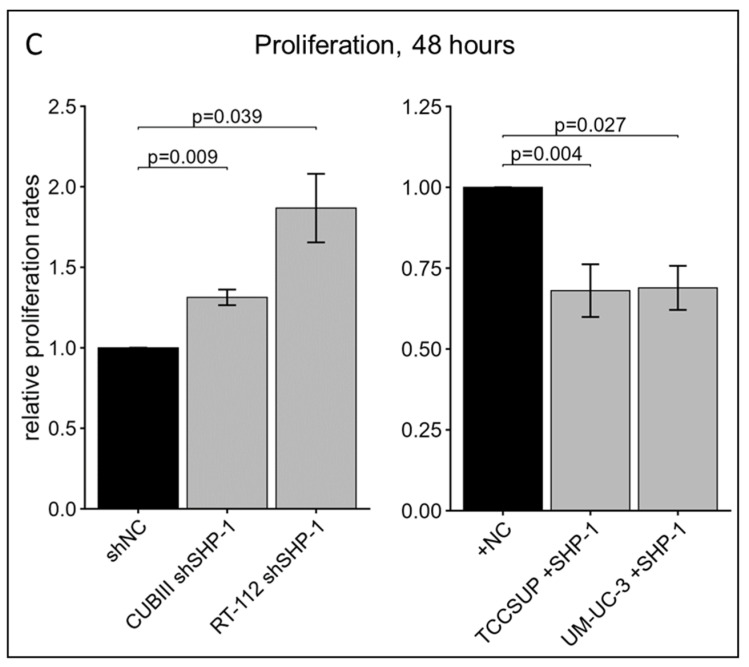
(**A**) SHP-1 protein expression following transduction with SHP-1 shRNA (shSHP-1) or SHP-1 cDNA (+SHP-1) and corresponding negative controls (NC): CUBIII n = 6, RT-112 n = 7, TCCSUP n = 4, UM-UC-3 n = 6. (**B**) Representative Western blot images: the first lane of each pair corresponds to SHP-1, and the second corresponds to the total protein detected for the same lane. Error bars represent the SEM. (**C**) Relative proliferation rates of BCa cells transduced with SHP-1 shRNA (shSHP-1) or SHP-1 cDNA (+SHP-1) and corresponding negative controls (NC): CUBIII n = 5, RT-112 n = 5, TCCSUP n = 9, UM-UC-3 n = 5. Error bars represent the SEM.

**Figure 5:**
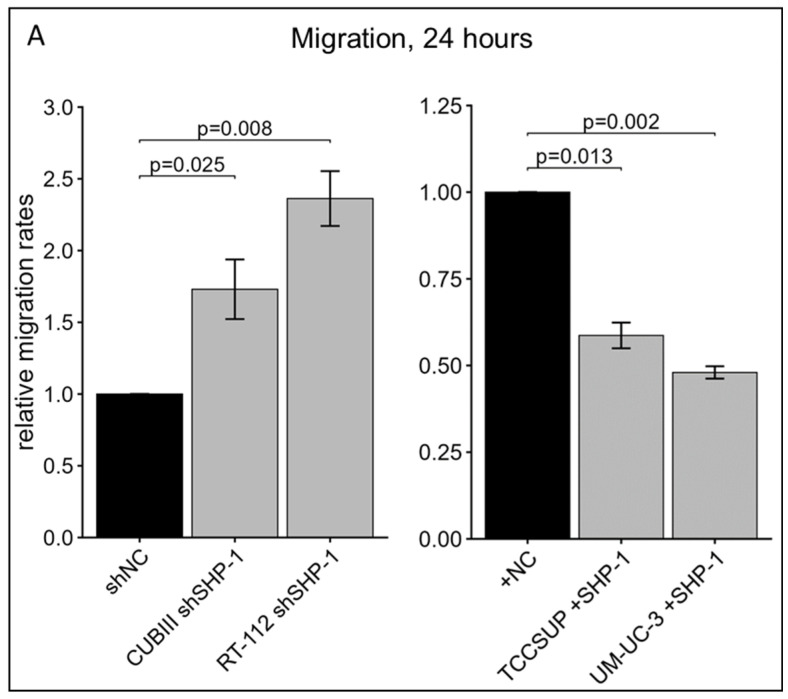

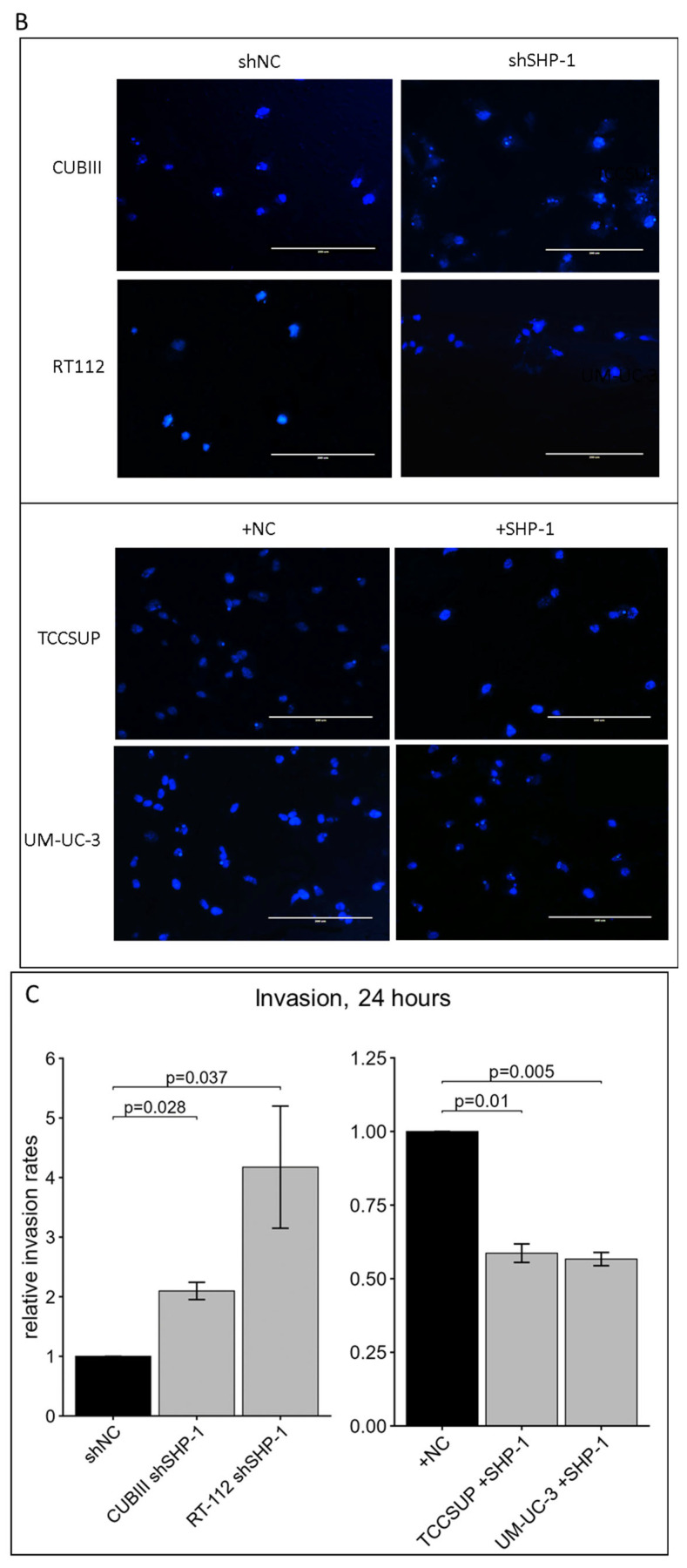

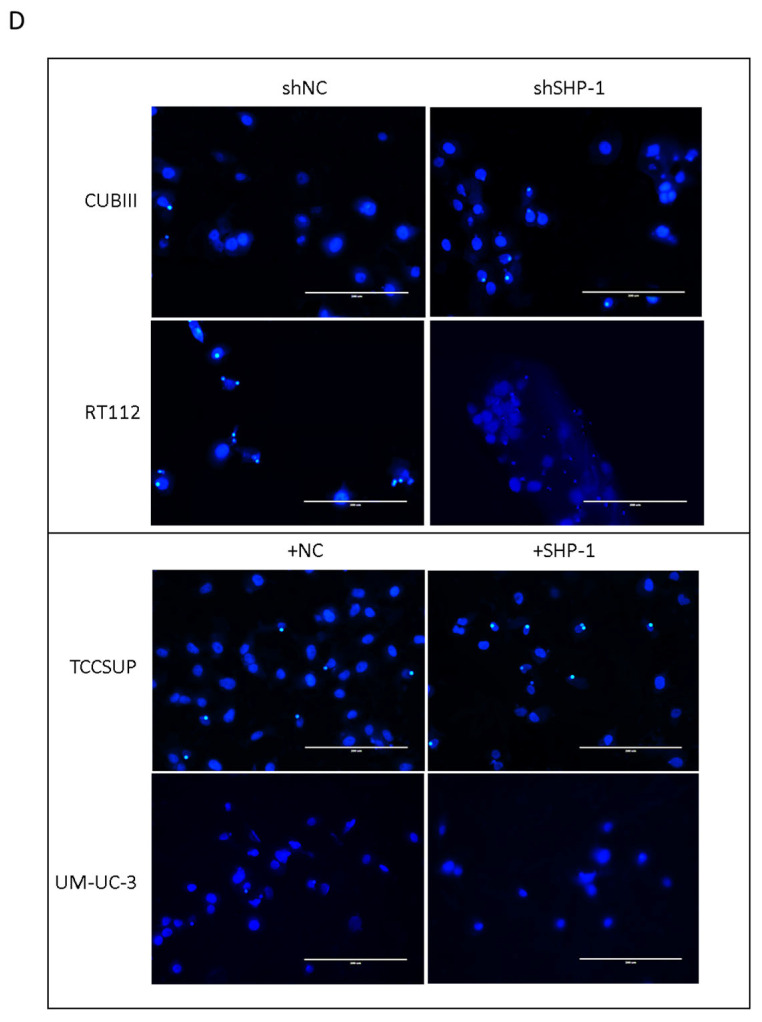
(**A**) Relative migration rates of BCa cells transduced with SHP-1 shRNA (shSHP-1) or SHP-1 cDNA (+SHP-1) and corresponding negative controls (NC): CUBIII n = 5, RT-112 n = 4, TCCSUP n = 3, UM-UC-3 n = 3. (**B**) Representative images from in vitro migration assays (scale bar = 200 μm). (**C**) Relative invasion rates of BCa cells transduced with SHP-1 shRNA (shSHP-1) or SHP-1 cDNA (+SHP-1) and corresponding negative controls (NC): CUBIII n = 3, RT-112 n = 5, TCCSUP n = 3, UM-UC-3 n = 3. (**D**) Representative images from in vitro invasion assays (scale bar = 200 μm). Error bars represent the SEM.

To establish a functional role for SHP-1, we modulated its endogenous expression using knockdown (shRNA) in high-expressing lines (CUBIII and RT-112) and stable over-expression in low-expressing lines (UM-UC-3 and TCCSUP), and then confirmed this via Western blot (Figure 4A,B). Functional assays demonstrated that SHP-1 exerts tumor-suppressive effects. Knockdown of SHP-1 significantly increased cell proliferation at 48 h, while SHP-1 restoration significantly attenuated proliferation (Figure 4C). In addition, in vitro assays showed that SHP-1 depletion led to a significant increase in migratory and invasive capacities. Conversely, restoration of SHP-1 expression significantly reduced these aggressive phenotypes (Figure 5).

### 3.4. SHP-1 Modulates Oncogenic Signaling and Suppresses the PI3K/Akt Pathway

**Figure 6:**
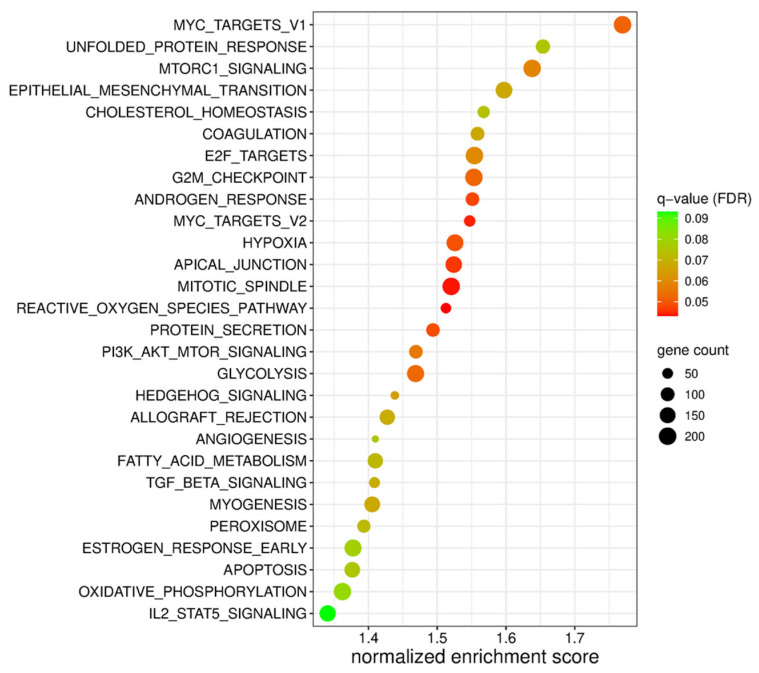
Significant gene sets resulting from GSEA of RNA sequence data comparing the transduced BCa lines with high-SHP-1-expressing lines versus low-SHP-1-expressing lines (q-value < 0.1).

NGS gene expression analysis and GSEA were performed to identify broad transcriptomic changes related to SHP-1 dysregulation. The mean read depth was 38.4 million per sample, with an average Q30 score of 92.3%. There were 41,440 unique genes detected. Comparing the high- versus low-expressing SHP-1 phenotypes, GSEA identified 28 of 50 hallmark gene sets as significantly enriched (FDR < 0.1) in several key oncogenic and metabolic pathways associated with EMT and cell cycle regulation (Figure 6).

**Figure 7:**
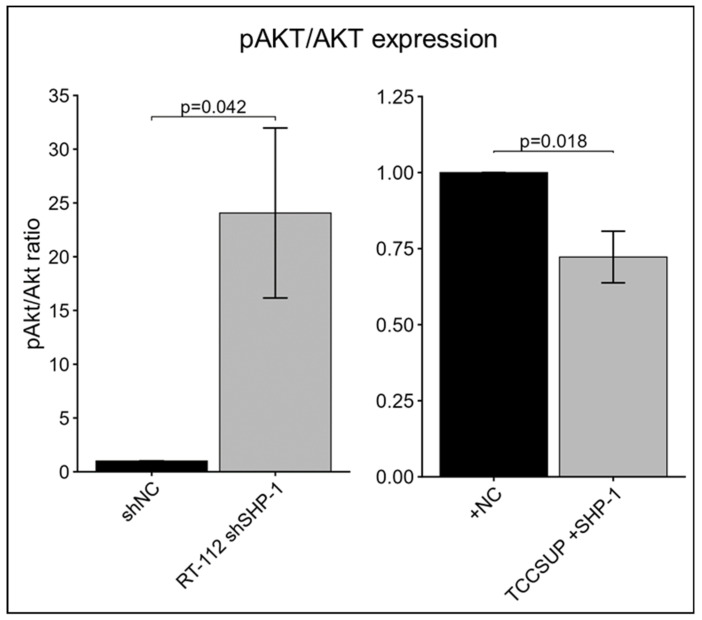

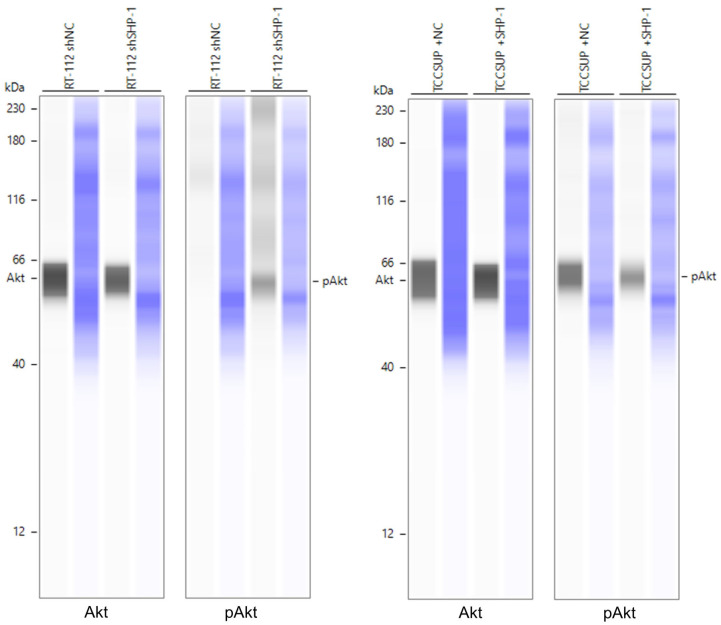
Relative expression of pAkt/Akt in BCa cells transduced with SHP-1 shRNA (shSHP-1) or SHP-1 cDNA (+SHP-1) and corresponding negative controls (NC): RT-112 n = 7 and TCCSUP n = 8. Representative Western blot images: the first lane of each pair corresponds to Akt or pAkt (ser473), and the second corresponds to the total protein detected for the same lane. Error bars represent the SEM.

To elucidate the potential mechanism by which SHP-1 inhibits BCa progression, we examined the PI3K/Akt signaling axis, a known driver of EMT and proliferation. Western blot analysis revealed that SHP-1 knockdown in RT-112 cells increased the pAkt/Akt ratio, whereas SHP-1 restoration in TCCSUP cells led to a significant decrease in Akt phosphorylation (Figure 7). In contrast, assessment of pAkt levels in CUB III and UM-UC-3 cells was inconsistent, with poor reproducibility observed across technical replicates, precluding reliable interpretation in these lines.

## 4. Discussion

SHP-1 is a well-known regulator of oncogenesis, and evidence supports its role in suppressing EMT in several tumor types. In this study, we provide evidence supporting its tumor-suppressive role in BCa. To the best of our knowledge, this is the first study to examine the relationship between EMT and BCa involving SHP-1 and to identify potential downstream targets and signaling pathways associated with SHP-1 dysregulation. Our findings extend previous reports on colorectal cancer [17], hepatocellular carcinoma [14], and breast cancer [15], suggesting that the suppressive function of SHP-1 in regard to EMT may be conserved across several tumor types. Studies have shown that SHP-1 constrains EMT by dephosphorylating key signaling molecules related to STAT3-, Akt-, and ERK-mediated transcriptional programs [34]. Downregulation of SHP-1 permits signaling that promotes the EMT phenotype. Our evidence suggests that SHP-1 loss correlates with EMT induction in BCa; SHP-1 expression is reduced in muscle-invasive disease, and SHP-1 inversely regulates proliferation, migration, and invasion. This is in line with findings from hepatocellular [14], breast [15], and colorectal [17] cancers, demonstrating that a loss of SHP-1 activity is associated with enhanced EMT signaling and aggressive tumor behavior.

The results presented here demonstrate that the loss of SHP-1 expression is associated with the EMT phenotype in BCa, which is a key mechanism underlying tumor invasion and metastasis. Elevated SHP-1 expression was positively correlated with increased levels of the epithelial marker E-cadherin and reduced expression of the mesenchymal markers N-cadherin and Vimentin. In contrast, decreased SHP-1 expression was associated with E-cadherin downregulation and upregulation of N-cadherin and Vimentin, changes that are characteristic of EMT induction. In addition, we observed that SHP-1 expression was significantly reduced in muscle-invasive BCa compared with in non-muscle-invasive disease or urothelium with NRD. The development of muscle invasion represents a critical step in BCa progression, and it is associated with poorer patient outcomes. Thus, a reduction in SHP-1 expression suggests a potential role for its loss in promoting tumor aggressiveness. Our functional analyses further indicate that SHP-1 inversely regulates key malignant behaviors in BCa cells, including proliferation, migration, and invasion. An increase in SHP-1 expression was associated with decreased cell proliferation, migration, and invasion capacities. In contrast, SHP-1 downregulation enhanced these aggressive phenotypes. These findings support the possibility that SHP-1 is associated with the regulation of BCa progression, potentially in relation to the EMT phenotype. Together, the results are compatible with, but do not establish, a tumor-suppressive role for SHP-1 in BCa.

Our GSEA of cells with altered SHP-1 expression revealed significant enrichment of EMT-related gene sets. Most notable was the enrichment of the EMT hallmark gene set, indicating a coordinated upregulation of mesenchymal markers and EMT-associated factors. For BCa, this coordinated enrichment of EMT is characterized by increased cell motility, invasiveness, and a heightened phenotypic plasticity that is associated with tumor aggressiveness [35]. In addition, enrichment of the apical junction gene set suggests regulation of cell–cell adhesion complexes. Alterations in apical junction components are a defining feature of EMT, as a disruption of junctions facilitates the loss of epithelial polarity and promotes cytoskeletal reorganization [36]. These gene set signatures therefore reflect coordinated restructuring of the epithelial architecture. We also observed significant enrichment of TGF-β signaling, which is a well-established driver of EMT [37]. Activation of this pathway is known to induce EMT through SMAD-dependent and SMAD-independent mechanisms, promoting transcriptional reprogramming toward mesenchymal gene expression [37]. Also, the enrichment of TGF-β signaling alongside EMT and junctional remodeling gene sets suggests that EMT-inducing pathways are actively engaged in this context. Collectively, these findings provide transcriptomic evidence that the observed phenotype is linked to TGF-β-mediated EMT activation and associated alterations in cell adhesion and structural organization.

Our GSEA further identified enrichment of gene sets for PI3K-Akt-mTOR signaling and MTORC1 signaling, suggesting that these pathways may mechanistically link SHP-1 modulation to EMT activation in BCa cells. The PI3K-Akt-mTOR axis is a central regulator of cell growth, survival, metabolism, and motility, and it has been widely implicated in the induction of EMT across several tumor types, including BCa [8]. Enrichment of these gene sets potentially indicates a coordinated transcriptional activation of signaling programs that promote mesenchymal transition and an aggressive tumor biology. Additionally, MTORC1 signaling has been linked to cytoskeletal remodeling, metabolic reprogramming, and enhanced protein synthesis required for sustained EMT phenotypes [38].

Aberrant activation of Akt signaling is a central driver of tumor progression and EMT in several cancers, largely through sustained phosphorylation of Akt at its activating residues [39]. Likewise, Akt phosphorylation has been implicated in an aggressive BCa phenotype [40]. In the context of SHP-1 regulation, downregulation of SHP-1 has been associated with increased Akt phosphorylation, thereby promoting EMT-associated phenotypes. Given that SHP-1 is a protein tyrosine phosphatase, its observed effect on Akt phosphorylation is likely indirect, potentially through dephosphorylation of upstream receptor tyrosine kinases or PI3K pathway components, which in turn modulate Akt activation, consistent with the changes in the pAkt levels observed in our model. Importantly, these transcriptomic findings are supported by our data demonstrating an increased pAkt/Akt ratio, which is consistent with enhanced Akt activation in BCa cell lines with decreased SHP-1 expression. These results indicate that SHP-1 could potentially be involved in the regulation of Akt activation in BCa and are in line with observations of a tumor-suppressive role in other malignancies. From a clinical perspective, increased pAkt signaling has been linked to poor prognosis and therapeutic resistance in BCa patients [41]. Given that SHP-1 is a protein tyrosine phosphatase with an established role in negatively regulating growth factor-mediated signaling, reduced SHP-1 activity may relieve inhibitory constraints on the PI3K-Akt pathway, thus facilitating downstream mTOR and MTORC1 activation. Collectively, these findings support a model in which SHP-1 downregulation promotes invasive signaling and contributes to EMT-driven progression in BCa cells.

From a clinical perspective, EMT is increasingly recognized as a key determinant of metastatic competence, immune evasion, and therapeutic resistance. The association between SHP-1 loss and EMT activation observed in other malignancies, together with our findings in BCa, highlights SHP-1 as a potential prognostic indicator of tumor aggressiveness [14,42]. Moreover, therapeutic strategies aimed at restoring or enhancing SHP-1 activity may represent a promising approach to suppress EMT and improve treatment responsiveness.

## 5. Conclusions

There are several limitations to this study. First, our findings rely primarily on in vitro models, which do not fully represent the complexity of the in vivo tumor environment. We note that the shRNA-mediated silencing achieved partial SHP-1 knockdown, and this incomplete suppression may underestimate the magnitude of the SHP-1-dependent effects observed in our assays. Therefore, their translational relevance to clinical settings needs to be further validated. The cell lines utilized in this study were not independently authenticated. However, they were expanded for a limited number of passages once thawed. Cell morphologies were routinely monitored and were consistent with expected features, and the EMT marker profiles were consistent with published reports for each line. In addition, the analysis of SHP-1 protein expression in bladder tissue was exploratory and quite limited in scope. The sample size was limited, and our tissue-based observations should be interpreted cautiously. In addition, it utilized NRD tissue in place of normal urothelium without a history of bladder cancer. Although NRD specimens are histologically free from detectable tumors, they may contain molecular alterations associated with the disease history, corresponding treatment, or field effects. As such, the observed differences in SHP-1 protein expression may differ from those in true normal urothelium and should be interpreted with caution. A larger independent cohort will be necessary to confirm the observed associations. In addition, the study population consisted exclusively of male subjects. However, TCGA expression data also demonstrated no statistically significant differences in SHP-1 gene expression between male and female BCa samples [24]. BCa has a higher prevalence in males, and it is known that sex-based biological differences exist; this limits the applicability of our findings and warrants future investigation in more diverse cohorts. Despite these limitations, and the preliminary nature of the tissue results, to the best of our knowledge, this study is the first report demonstrating that SHP-1 protein expression is associated with BCa aggressiveness, as well as the first known report indicating that SHP-1 is related to EMT in BCa. This novel observation provides a foundation for future mechanistic and clinical studies to further define the role of SHP-1 in BCa pathology.

## Figures and Tables

**Table 1:** Patient demographics for the samples used in this study. Urothelial tissue with no residual disease (NRD), non-muscle-invasive tumors ≤ T1 (NMI), and muscle-invasive tumors ≥ T2 (MI).

	NRD (n = 10)	NMI ≤ T1 (n = 8)	MI ≥ T2 (n = 8)	*p*-Value
median age, years (range)	77 (65–84)	68 (59–83)	76 (69–84)	0.07
smoking history, n (%)	6 (60)	5 (62.5)	7 (87.5)	0.563
prior BCG treatment, n (%)	3 (30)	2 (25)	3 (37.5)	1

## Data Availability

The data presented in this study are available on request from the corresponding author.
